# The effect of epigenetic reprogramming using MI192 HDAC inhibitor on enhancing the osteogenesis of human adipose-derived stem cells *in vitro*

**DOI:** 10.1042/BSR20221635

**Published:** 2023-04-28

**Authors:** Wei Lu, Kun Ji, Liam Lawlor, Sushmita Saha, Aiden Hempshall, Yan Jin, Xuebin B. Yang

**Affiliations:** 1 Department of Prosthodontics, Nanjing Stomatological Hospital, Affiliated Hospital of Medical School, Nanjing University, Nanjing, China; 2 Biomaterials and Tissue Engineering Group, School of Dentistry, University of Leeds, Leeds LS9 7TF, U.K.; 3 Department of Paediatric Dentistry, Nanjing Stomatological Hospital, Affiliated Hospital of Medical School, Nanjing University, Nanjing, China; 4 Molecular Innovation, Diversity and Automated Synthesis (MIDAS) Centre, School of Chemistry, University of Leeds, Leeds LS9 7TF, U.K.; 5 State Key Laboratory of Military Stomatology and National Clinical Research, Center for Oral Diseases and Shaanxi Key Laboratory of Oral Diseases, Center for Tissue Engineering, Fourth Military Medical University, Xi'an, Shaanxi, 710032, China

**Keywords:** adipose-derived stem cells, cell cycle, histone deacetylase inhibitor, MI192, osteogenic differentiation, valproic acid

## Abstract

The ability to control stem cell function is the key to stem cell-based therapy and living tissue regeneration. In natural conditions, histone deacetylases (HDAC) are regarded as the important defining epigenetic reprogramming for stem cell differentiation. To date, human adipose-derived stem cells (hADSCs) have been widely utilised for bone tissue engineering applications. The present study aimed to examine the effect of a novel HDAC2&3-selective inhibitor, MI192, on hADSCs epigenetic reprogramming for regulating its osteogenic potential *in vitro*. The results confirmed that MI192 treatment reduced the hADSCs viability in a time and dose-dependent manner. The optimal concentration and pre-treatment time of MI192 for hADSCs osteogenic induction was 30 μM and 2 days representatively. A quantitative biochemical assay confirmed that the pre-treatment with MI192 (30 μM) for 2 days significantly enhanced hADSCs alkaline phosphatase (ALP) specific activity (*P*<0.05) compared with that of the valproic acid (VPA) pre-treatment group. Real-time PCR analysis revealed that MI192 pre-treatment up-regulated hADSCs gene expressions of osteogenic markers (e.g., Runx2, Col1, and OCN) under the osteogenic induction. DNA flow cytometric analysis indicated that two days’ pre-treatment with MI192 (30 μM) resulted in G2/M arrest in hADSCs and this G2/M arrest was reversible. Our results suggest that MI192 is capable of epigenetic reprogramming of hADSCs via HDAC inhibition for controlling the cell cycle, resulting in enhancing hADSCs osteogenic differentiation, which indicates the potential of using MI192 for promoting bone tissue regeneration.

## Introduction

Over the last decade, skeletal tissue engineering has become one of the most popular emerging technologies for tackling the clinical need in orthopaedics [1,2]. Due to the ethical concerns of using embryonic stem cells for clinical treatment [3], mesenchymal stem cells (MSCs) are generally accepted as one of the most promising cell candidates for bone tissue engineering [4,5]. However, to date, the inefficiency of stem cell therapies in clinical trials has led to the search for alternative approaches for meeting the clinical requirement [6,7]. Therefore, the ability to control stem cells' lineage-specific differentiation is fundamental for the future of tissue regeneration [8,9]. In natural human development, it is well known that epigenetic modifications (such as the acetylation process of histone and non-histone proteins) can control DNA accessibility and chromatin structure, affecting cell gene expression patterns and, consequently, cell functions. The cell’s epigenetic makeup can be formed in response to the cell’s environment and is known to be passed down across cell generations. Thus, influencing a cell’s epigenetic makeup could be a safe way of controlling stem cell behaviour.

It is well known that both histone acetyltransferases (HATs) and histone deacetylases (HDACs) dynamically regulate the acetylation process of cells. HAT results predominantly in the presence of the acetylated lysine in the histone tails, ‘opening’ the structure of human chromatin, ‘allowing’ and/or ‘activating’ gene transcription. In contrast, HDAC activity removes the acetyl groups, which leads to a more ‘condensed’ and/or ‘closed’ chromatin structure, which ‘stops’ gene transcription (e.g. gene silencing) [10–12]. In general, HDAC has been clarified as four categories: Class I, including HDAC-1,-2,-3, and -8, are primarily localised in nuclear; Class IIa (e.g. HDAC-4,-5,-7, and-9) are between the nucleus and cytoplasm, and Class IIb (HDAC-6 and -10) are localised mainly in the cytoplasm. Class III HDACs (sirtuins, SIRT1–SIRT7) are structurally different [13]. Of note, HDAC3 is different from the other Class I HDACs, as it can appear in both the cell’s nucleus and the cytoplasm of cells. HDAC3 forms a well-characterised protein complex, which can be recruited to specific promoters leading to the deacetylation of histones [14]. HDAC3 can also regulate gene expression and inflammatory response by interacting with other Class II HDACs [15,16]. In the cytoplasm, HDAC3’s role is still not completely clear, and it is thought to be involved in signal transduction and/or mitosis [17,18]. Lu et al. (2018) reported that HDAC3 has the potential to promote liver regeneration via the activator of the transcription 3 (STAT3) signalling pathway [19].

It is well known that the histone deacetylase inhibitors (HDACis) can readjust the balance between the HAT and HDAC enzyme's activities in both tumour and normal cells, resulting in the accumulation of acetylated histone and non-histone proteins. This, in turn, alters the regulation of the cell cycle, cellular gene expression, migration, proliferation, differentiation, angiogenesis, apoptosis, and many other cellular processes [12,20]. There is a range of HDACs available in the market that has been used for various clinical treatments for cancers and non-cancerous diseases [21–23]. However, most studies related to HDACs/HDACis are in the field of cancer therapy. There is strong evidence to show that HDACis arrest tumour cell growth and prompts apoptosis [24,25].

Over the last few years, HDACis have been reported to inhibit human MSC proliferation but also promote osteogenic differentiation and osteoblast maturation *in vitro* [22,26–28]. For example, trichostatin A (TSA), a pan-HDAC inhibitor, has been shown to accelerate matrix mineralisation in different types of MSCs [29,30]. Sodium butyrate (NaB) can enhance periodontal ligament fibroblasts' osteogenic differentiation [31]. The other reports showed that TSA, Entinostat (MS-275), and VPA could up-regulate osteogenic the gene expression of osteogenic markers [32–34].

Karantzali and co-workers (2008) reported that the inhibition of HDACis can diminish stem cell self-renewal. This may be due to the inhibition of HDACs can up-regulate stem cells to the expression of different differentiation markers, resulting in the acceleration of stem cell lineage-specific differentiation [35–39]. However, it was reported that VPA could promote normal hematopoietic stem cell proliferation and self-renewal [40,41]. Specifically, HDAC3 regulated several non-histoneproteins, including nuclear factor-κB (NF-κB) subunit RelA, cell cycle inhibitor p21 and p53 [42,43]. Although the full mechanisms of how the HDACis regulate stem cell lineage-specific differentiations are still unclear. However, the potential to utilise HDACis to control stem cell fate is evident [26,38,44].

MI192 is a novel benzamide family HDAC2 and HDAC3-selective inhibitor [45,46]. It has a terminal benzamide structure that is known to chelate Zn^2+^ ions, which can be found at the HDAC active site [14]. In our previous study, we showed that the MI192 enhanced the human dental pulp stromal cells’ osteogenic differentiation capacity [47]. Several studies showed that MI192 has dose-dependent inhibition for interleukin-6 (IL-6), and peripheral blood mononuclear cells (PBMC) in rheumatoid arthritis (RA) patients but not in healthy control [45,48,49]. High concentrations of MI192 can inhibit tumour necrosis factor (TNF) production in cancerous cells [49]. However, the effect of MI192 on human adipose-derived MSC’s osteogenic potential has not been reported.

To date, human bone marrow has been accepted as one of the most popular stem cell sources for tissue engineering [6,50]. Some papers directly compared MSCs derived from bone marrow with that from the adipose tissue, showing that human adipose tissue-derived stem cells (hADSCs) are higher proliferative, although bone marrow MSCs may have greater osteogenic and chondrogenic potential [51–53], Importantly, adipose tissue is a more readily available and highly accessible stem cell source for scalable tissue engineering purposes [54–60].

Thus, the present study aimed to investigate the potential of using MI192, the novel HDAC2/3-selective inhibitor, to promote osteogenic differentiation of hADSCs *in vitro*.

## Materials and methods

### Cell culture and reagents

HDAC2/3-selective inhibitor, N-(2-aminophenyl) benzamide derivatives known as MI192, was produced at Prof Ronald Grigg’s group, University of Leeds. PA sodium salt was purchased from Sigma (Cat No: P4543). MI192 was solubilised in dimethyl sulfoxide (DMSO) (Sigma-Aldrich, U.K.), and the final concentration of DMSO in the culture medium was lower than 0.1%. VPA was dissolved in 1× phosphate-buffered saline (PBS). Human adipose-derived stem cells (hADSCs) were purchased from Invitrogen (Catalogue no. R7788-115) following isolation from human adipose tissue collected during liposuction procedures. Before cryopreservation, the hADSCs were expanded for one passage in MesenPRO RS™ medium. The hADSCs were checked by the manufacturer with flow cytometry to confirm that the cells expressed positively for CD29, CD44, CD73, CD90, CD105, and CD166 but negatively for CD14, CD31, CD45, and Lin1 [61]. The multipotential identity of the hADSCs used in the present study was confirmed using tri-lineage differentiation methods and flow cytometry analysis (data were shown in Supplementary Figures S1 and S2). The results were consistent with the literature [25,62]. The hADSCs were expanded in basal medium, which contain α-modified minimal essential medium (αMEM) (Lonza BioWhittaker, Belgium) supplemented with 5% fetal bovine serum (FBS) (BioSera, Sussex, UK) and 1% penicillin-streptomycin (Sigma, P0781). hADSCs were passaged when they reached about 80% confluence. Passage 5 (P5) cells were then used for all experiments.

### Culture of hADSCs with different concentrations of MI192

hADSCs (P5) were seeded in a 12-well plate (2000/cm^2^) and cultured in basal medium for one day. Then, hADSCs in the exponential growth phase were treated with different concentrations of MI192 (1, 10, and 100 µM) in basal medium (*n*=3) for up to 5 days. Osteogenic induction medium (OS) (basal medium in supplement with 100 nM dexamethasone, 10 mM b-glycerophosphate, and 0.2 mM ascorbic acid-2-phosphate) [63,64] culture was used as the positive control, and basal medium culture was used as the negative control. At different time points (e.g., days 1, 3, and 5), cell phenotype change, proliferation, and osteogenic differentiation were assessed under inverted microscopy or via alkaline phosphatase (ALP) staining. The population doubling time was calculated as previously reported [65].

### Measuring the cytotoxic effect of MI192 on hADSCs

hADSCs (P5) were seeded in 96-well plates (2000/cm^2^) and cultured in basal medium for one day. Then, hADSCs were treated with different concentrations of MI192 (1–50 µM) in basal medium (*n*=3) for 1, 2, and 3 days. Basal medium alone (untreated wells) was used as the control. The CytoTox 96® Non-Radioactive Cytotoxicity Assay kit (Promega, Cat. no: G9260) was used according to the manufacturer’s instructions. Briefly, the prepared reagent (100 µl per well) was added directly to the medium of the plate (including the control wells). The plate was then incubated for 30 min at 37°C, and the fluorescence was then measured in a Varioskan Flash Multimode Microplate Reader (Model 3001, Thermo Scientific) by exciting the samples at 488 nm with an emission reading set at 520 nm.

### Optimisation of the MI192 concentration for pre-treatment of hADSCs undergoing osteogenic induction

hADSCs (P5) were seeded in a 12-well plate (2000/cm^2^) as described previously and cultured in a basal medium for one day. A lower range of MI192 concentrations (10, 20, 30, and 40 µM) and a higher range of MI192 concentrations (e.g., 50, 80, 100, 150, and 200 µM) was used to pre-treat the hADSCs for 2 days. Old media was then discarded, and the cells were cultured in OS for a further 5 days. Osteogenic differentiation of hADSCs was confirmed by ALP staining.

### Optimisation of the best time of MI192 pre-treatment for hADSCs undergoing osteogenic induction

After one day of seeding in a 24-well plate (as described previously), hADSCs (P5) were pre-treated with the optimal concentration (30 µM) of MI192 for 1, 2, and 4 days. Then, the old media were discarded, and the cells were cultured in OS for a further 5 days. Osteogenic differentiation of hADSCs was confirmed by ALP staining. ALP staining on another batch of hADSCs pre-treated with MI192 for 2 days and osteogenic induction for 5 days (data were shown in Supplementary Figure S3).

### Optimisation of the combination of pre-treatment period and concentration of MI192 for osteogenic induction – quantitative ALP-specific activity

hADSCs (P5) were seeded (2000/cm^2^) in a 24-well plate as described above and cultured in a basal medium for one day. A range of MI192 concentrations (20, 30, 40, and 50 µM) was used to pre-treat the hADSCs for 1 and 2 days. Then, the old media were replaced with OS for a further 7 days. The cells were then lysed for a quantitative alkaline phosphatase specific activity (ALPSA) assay using the 4-nitrophenyl colourimetric phosphate liquid system (Sigma-Aldrich, Cat. no: 4264-83-9) as previously described [50]. The total DNA quantity was assessed by ultrasensitive fluorescent nucleic acid stain Quant-iT™ PicoGreen® DNA quantification assay (Invitrogen, Life Technologies, Cat. no: P7581). ALPSA for each well was calculated using the total ALP quantity divided by the total DNA content of the same well (nmol ALP/hour/µg of DNA) [50].

### Cell cycle analysis

hADSCs (P5) in the exponential growth phase were pre-treated with 30 and 80 µM of MI192 for 2 days. A commercial product – valproic acid (VPA, 2 mM) [66] and basal medium culture were used as the controls (*n*=3). The hADSCs were collected separately and washed twice with 1× PBS prior to being fixed in cold 70% alcohol at 4°C for 24 h. Then, the cells were stained with propidium iodide (100 µg/ml; Sigma, St Louis, MO) at 4°C for 30 min and subjected to Elite ESP flow cytometry (Beckman Coulter Inc, Fullerton, CA). Cell cycle analysis was performed on at least three independent synchronisation experiments [67].

### Real-time RT-PCR

hADSCs (P5) were seeded in a T25 flask (2 × 10^5^ cells per flask) and cultured in basal medium for one day at 37°C, 5% CO_2_. Then, hADSCs were pre-treated with 30 µM MI192 and 2 mM VPA in basal medium for a further 2 days (*n*=3). Basal medium alone was used as a control. The old media were then discarded, and the cells were cultured in OS for up to 15 days. At different time points (days 0, 5, 10, and 15), the cells were collected, and total RNA was extracted using TRIzol reagent (Invitrogen Life Technology, Carlsbad, CA). First-strand cDNA syntheses were performed according to the manufacturer’s protocol. Quantitect Sybr Green Kit (Toyobo, Osaka, Japan) and ABI Prism 7500 Sequence Detection System (Applied Biosystems) were used for Real-Time PCR. Primer sequences used were:

1. Runt-related transcription factor 2 (*RUNX2*)(GenBank accession no. NM004348.3): sense, 5′-CACTGGCGCTGCAACAAGA-3′; antisense, 5′-CATTCCGGAGCTCAGCAGAATAA-3′.
2. Collagen typeI (*COL1*)(GenBank accession no. NM000088): sense, 5′-CCCGGGTTTCAGAGACAACTTC-3′; antisense, 5′-TCCACATGCTTTATTCCAGCAATC-3′.
3. Osteocalcin (*OCN*)(GenBank accession no. NM000711):sense, 5′-CCCAGGCGCTACCTGTATCAA-3′; antisense, 5′-GGTCAGCCAACTCGTCACAGTC-3′.
4. *β-Actin* (Gen Bank accession no. NM001101):sense, 5′-TGGCACCCAGCACAATGAA-3′;antisense, 5′-CTAAGTCATAGTCCGCCTAGAAGCA-3′.

*β-Actin* was used as an internal control. Amplification occurred after denaturing at 95°C for 30 s, followed by 35 cycles at 95°C for 10 s and 64°C for 30 s. The gene expression levels were normalised according to the level of *β-actin* expression.

hADSCs were pre-treated with 30 µM MI192 for 2 and 5 days, and relative mRNA expression of HDAC2 and HDAC3 in hADSCs was examined (data were shown in Supplementary Figure S4). Primer sequences used were: (1) histone deacetylase 2 (HDAC2): sense, 5′- CTGCTACTACTACGACGGTGAT-3′; antisense, 5′- CAGTGGCTTTATGGGGCCT-3′. (2) histone deacetylase 3 (HDAC3): sense, 5′- AGTTCTGCTCGCGTTACACA-3′; antisense, 5′- CAGAAGCCAGAGGCCTCAAA-3′.

### ALP staining

The cells were fixed in 98% ethanol for 30 min before being stained using an alkaline phosphatase (ALP) staining kit (Naphthol AS-MX phosphate alkaline solution, Sigma 855; Fast Violet B salt, Sigma 201596-5G).

### HDAC activity

Cells were cultured in one 96-well plate (1 × 10^4^ cells per well) in basal medium containing MI192 at a range of concentrations (1, 10, 30 and 50 µM). Basal medium alone was used as the control. At 48 h, the HDAC activity in the cells was measured post-treatment immediately using an InSitu HDAC Activity Fluorometric Assay Kit (Cambridge BioSciences, Cambridge, U.K.), according to the manufacturer’s instructions as per our previous study [47] (data were shown in Supplementary Figure S5).

### Western blot

Proteins from cells were lysed with a RIPA lysis buffer containing 1 mM PMSF (Beyotime), referring to the manufacturer’s instruction. Then, the concentration was measured by BCA Protein Assay Kit (Beyotime). The proteins were separated by 10% sodium dodecyl sulfate (SDS) polyacrylamide gels and transferred to polyvinylidene difluoride membranes (Sigma-Aldrich). The membranes were blocked in 6% skim milk for 2 h and then incubated with primary antibodies at 4°C overnight and finally with the secondary antibody (1:6000; Bioworld, Nanjing, China) for 1 h. The following primary antibodies were used: anti-β-actin (1: 6000; Proteintech™, WuHan, China), anti-RUNX2 (1:1000; Cell Signaling Technology, MA, U.S.A.), anti-osteocalcin (OCN) (1:1000, Proteintech™). The visualisation of the protein bands was used with an enhanced chemiluminescence kit (Beyotime) (the full uncropped and unedited versions of the Western blot were shown in Supplementary Figure S6).

### Alizarin red staining

The cells were fixed overnight at 4°C in 10% neutral buffered formalin and rinsed with tap water several times. After bleaching pigmentation with 3% H_2_O_2_ solution, the cells were stained with 1 mg/ml alizarin red stain/1% KOH and then sequentially washed with 20% glycerol/1% KOH, 40% glycerol/1% KOH, and 60% glycerol/1% KOH. Images of stained cells were obtained using a stereo microscope (SMZ1500) (Nikon, Tokyo, Japan). Mineralisation was calculated using the ImageJ densitometry program.

### Statistics

Values are expressed as mean ± SD. Experiments were performed at least three times, and the results of representative experiments are presented except where otherwise indicated. Statistical analysis was performed by analysis of variance and an independent samples *t*-test with SPSS software (v16.0) or using ANOVA multiple comparisons test with Tukey modification. *P*<0.05 is considered a significant difference.

## Results

### MI-192 was cytotoxic at high concentrations and inhibited hADSCs proliferation

**Figure 1:**
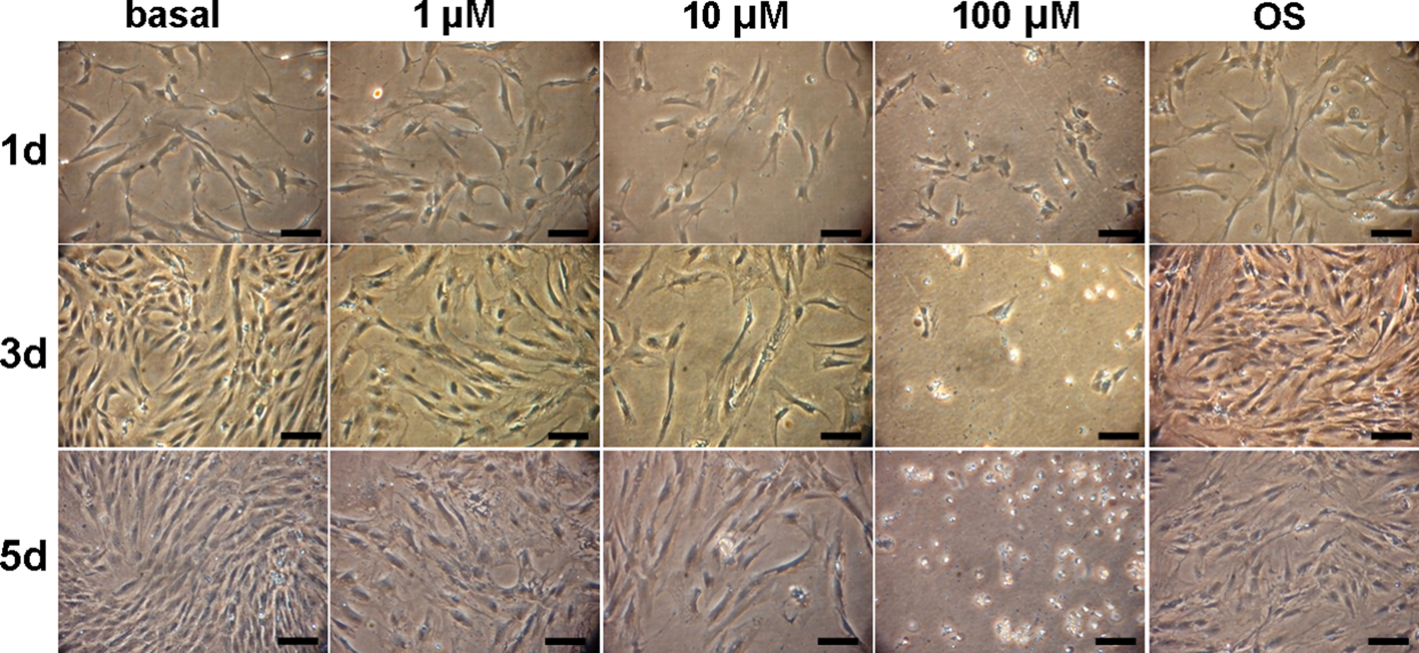
Morphological changes of hADSCs after 5 d MI192 treatment MI192 were tested for their ability to inhibit the proliferation of hADSCs. MI192 prevented cell growth in a concentration-dependent manner, although high concentrations are required to achieve a maximal effect. However, there was no significant difference in cell proliferation between the basal medium group and osteogenic medium group; scale bars: 50 μm.

This batch of hADSCs’ mean doubling time is 42 h in basal media culture and 50 h at early stages in osteogenic medium culture. The population doubling time of hADSCs in the presence of 1, or 10 µM of MI192 were 80 and 90 h. On day one, there was no significant difference in cell density between the lower concentration of MI192 (1 and 10 µM) compared with both control groups. On day 3 and 5, the 1 and 10 µM groups showed that the reduction of cell density was dose-dependent. In contrast, the higher concentrations (e.g., 100 µM) MI192 appeared to be cytotoxic to the cells from day 1, an effect which became more severe/obvious on day 3 and 5 (Figure 1).

**Figure 2:**
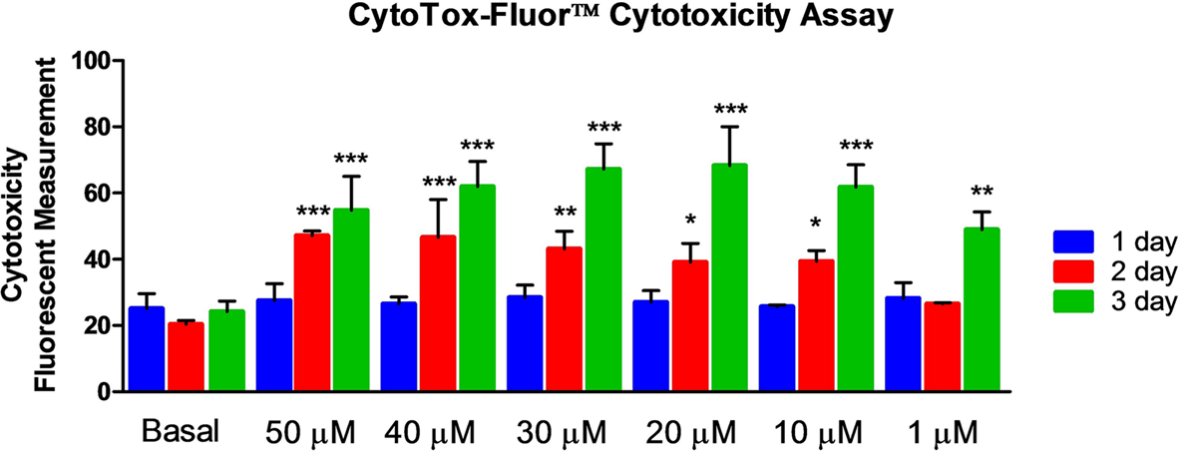
Cytotoxicity of MI192 on hADSCs assessed with CytoTox-Fluor™ cytotoxicity assay One day of pre-treatment with MI192 (1–50 µM), there were no statistically significant (*P*>0.05) between the test groups and the basal medium control group. However, after 2 days of pre-treatment with MI192, the 50 µM and 40 µM groups showed extremely statistically significant (*P*≤0.001) cytotoxic compared with the basal medium control. There were also statistically significant cytotoxic in the lower concentrations, with 30 µM (*P*≤0.01), 20 and 10 µM (*P*≤0.05) compared with the control group. There was no significant difference between the 1 µM MI192 group and the control group (*P*>0.05). After 3 days of pre-treatment, all higher concentrations of MI192 (10–50 µM) groups showed extremely significant (*P*≤0.001) cytotoxic compared with the control group. Even 1 µM MI192 group was also significantly more cytotoxic than the control group (*P*≤0.01). *P≤0.05, **P≤0.01, and ***P≤0.001.

The cytotoxicity of MI192 was also confirmed with Cyto Tox-Fluor™ cytotoxicity assay, which showed that after 1 day of pre-treatment with MI192 (1, 10, 20, 30, 40, and 50 µM), there was no statistically significant difference (*P*>0.05) between the test groups and the basal medium group (Figure 2). However, after 2 days of pre-treatment with MI192, the 50 and 40 µM groups showed an extremely statistically significant difference (*P*≤0.001) with more cytotoxic to the cells compared with that of the basal medium group. There were also statistically significant cytotoxic in the lower concentrations, with 30 µM (*P*≤0.01), 20 and 10 µM (*P*≤0.05) compared with the control group. There was no significant difference between the 1 µM of MI192 group and the basal medium group (*P*>0.05). After 3 days of pre-treatment, all higher concentrations of MI192 (10–50 µM) groups showed extremely significant (*P*≤0.001) cytotoxic compared with the control group. Even 1 µM of MI192 group was also significantly more cytotoxic than the basal medium group (*P*≤0.01).

Treatment with 1*–*50 µM MI192 for 48 h led to a significant reduction in hADSCs HDAC activity compared with that of the untreated cells in a dose-dependent manner (*P*≤0.001) (data were shown in Supplementary Figure S5).

### High concentration of MI192 inhibited hADSCs osteogenic differentiation

**Figure 3:**
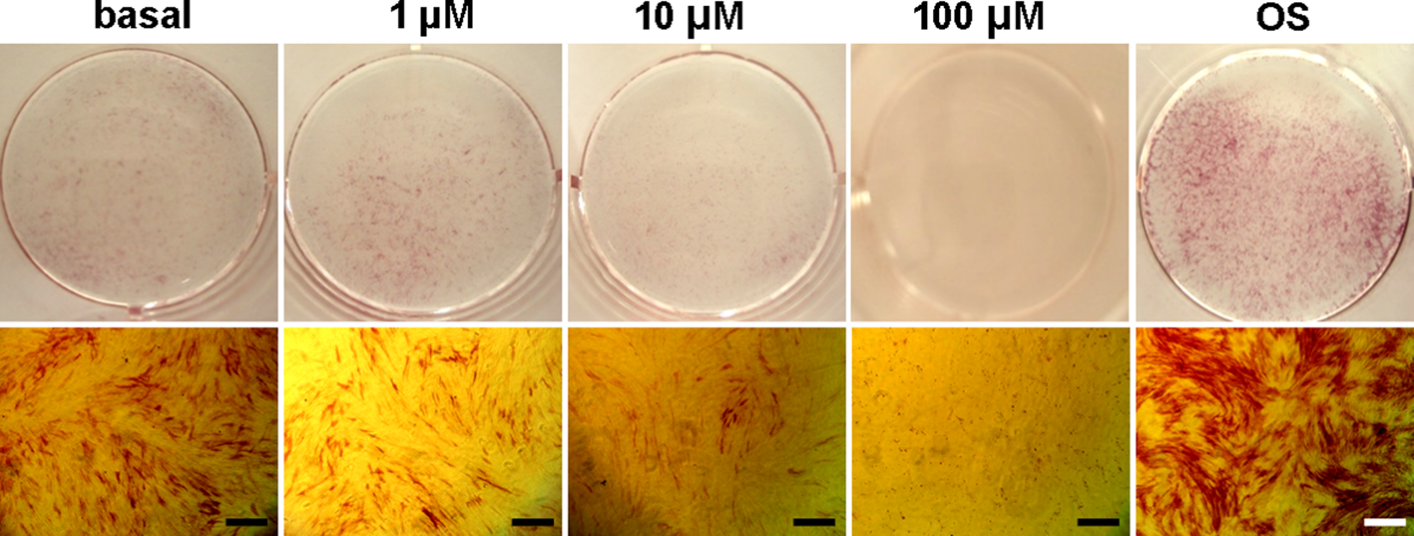
Effect of MI192 on osteogenic differentiation of hADSCs Through 5 days of MI192 effect at three concentrations (1, 10, and 100 µM), as well as basal medium and osteogenic medium induction, ALP staining showed that MI192 decreases self-osteogenic differentiation of hADSCs; scale bars: 50 μm.

After 5 days of culture with different concentrations of MI192 (1, 10, and 100 µM) in basal medium, ALP staining showed that 1, 10, and 100 µM of MI192 group showed reduced ALP staining compared with the OS positive control group, and the basal medium negative control group. OS positive control group showed an enhanced ALP staining compared with basal medium group. There was no positive ALP staining in the 100 µM of MI192 group (Figure 3).

### The optimal concentration of MI192 pre-treatment for osteogenic induction – ALP staining

**Figure 4:**
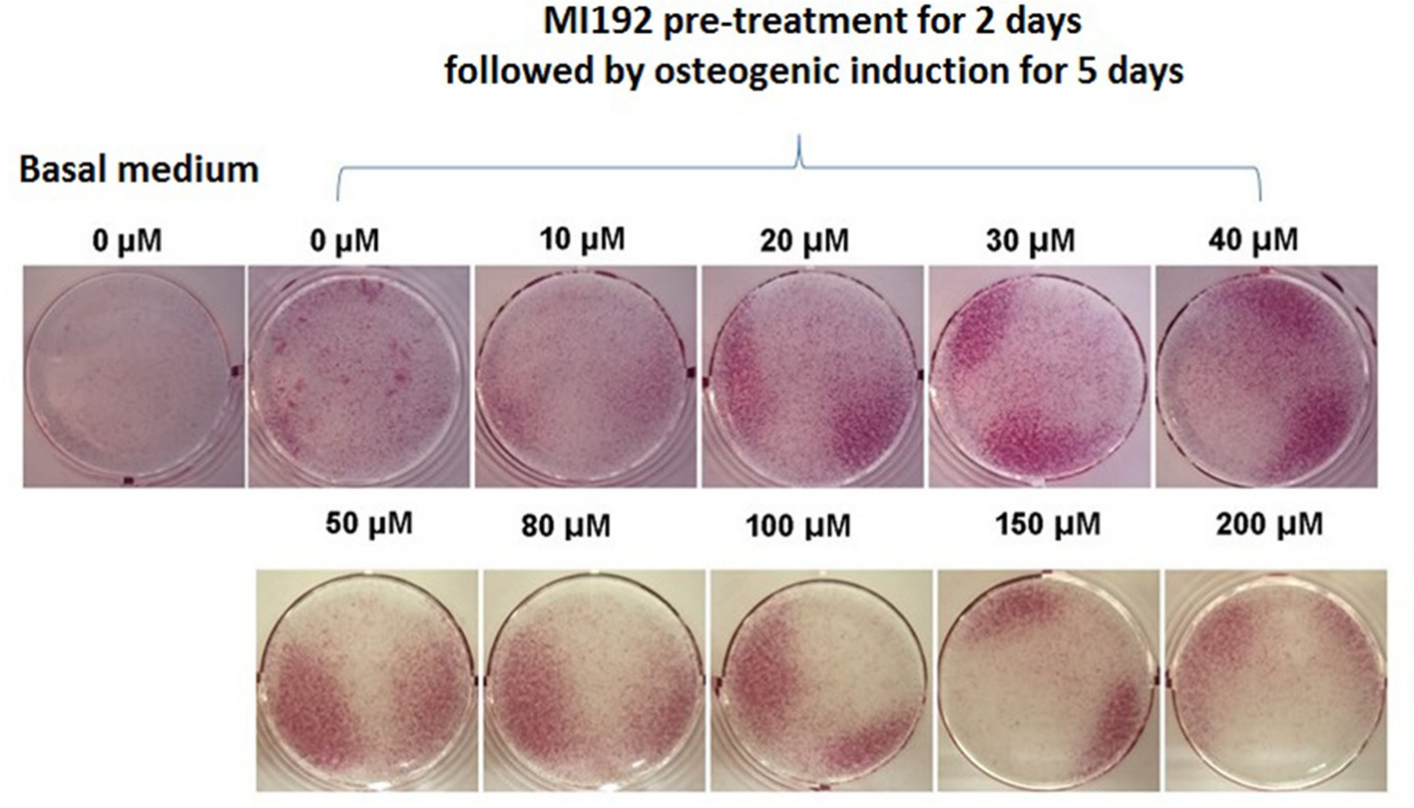
Best concentration of MI192 pre-treatment for osteogenic induction of hADSCs After 2 days of pre-treatment with different concentrations of MI192 in basal medium followed by 5 days of osteogenic culture, 20–200 µM groups enhanced ALP positive staining with the highest staining between 30 and 50 µM. Considering the maximum osteogenic effect, with the minimum side effects, 30 µM was selected as the optimal concentration of MI192 for the pre-treatment.

Since the side effects (e.g., cytotoxicity) of MI192 were correlated to the culture period, this experiment aimed to optimise the concentration of MI192 for pre-treatment of hADSCs. After 2 days of pre-treatment with different concentrations of MI192 in basal medium followed by 5 days of osteogenic culture, 20–200 µM groups enhanced ALP positive staining with the highest staining between 30 and 50 µM. A higher concentration of MI192 (>50 µM) showed some signs of cytotoxicity and reduction of cell densities (Figure 4). Considering the maximum osteogenic effect, with the minimum side effects, 30 µM was selected as the optimal concentration of MI192 for the pre-treatment.

### The optimal pre-treatment period with MI192 for osteogenic induction – ALP staining

**Figure 5:**
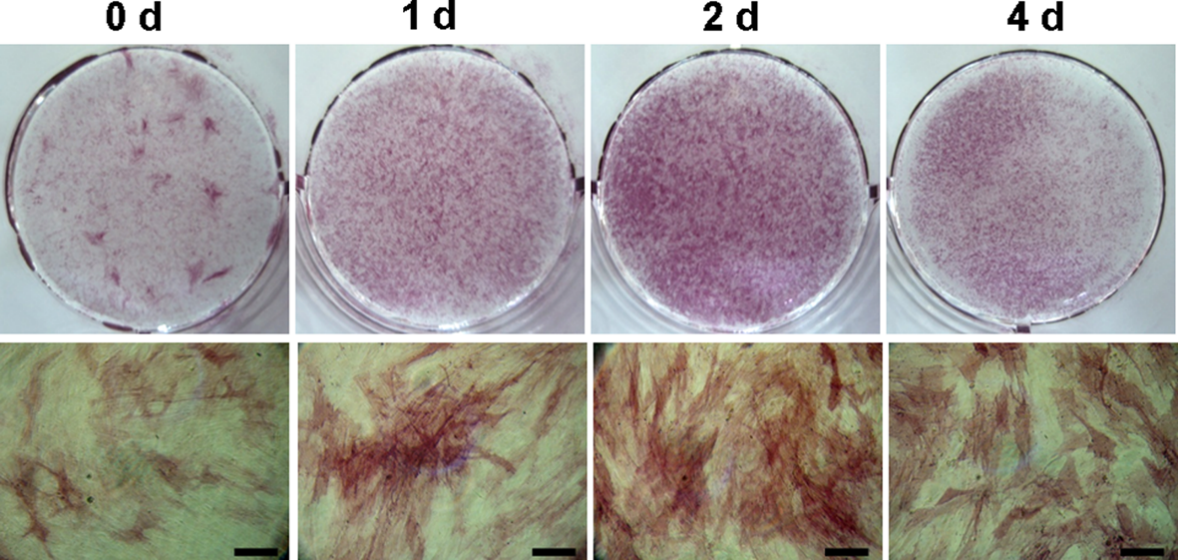
Best period for pre-treatment of MI192 for osteogenic induction of hADSCs MI192 (30 µM) was used for pre-treatment for 1, 2, and 4 days, and the induction of osteogenic differentiation of hADSCs was done without the addition of MI192. Osteogenic differentiation was estimated by alkaline phosphatase (ALP) staining after 5 days. The pre-treatment of MI192 for 2 days markedly increased osteogenic differentiation, and the pre-treatment for 1 day or 4 days was less than 2 days.

To determine the optimum period of the pre-treatment of MI192, 30 µM of MI192 was used to pre-treat the hADSCs for 1, 2, or 4 days prior to being cultured in OS for a further 5 days. ALP staining showed that the group that was pre-treated with 30 µM of MI192 for 2 days markedly increased the ALP stain intensity compared with that in the groups that were pre-treated for 1 day or 4 days (Figure 5). Therefore, hADSCs were pre-treated with 30 µM of MI192 for 2 days were used as the optimal method for the final experiments.

### The optimal pre-treatment period and concentration of MI192 for osteogenic induction – ALP-specific activity

**Figure 6:**
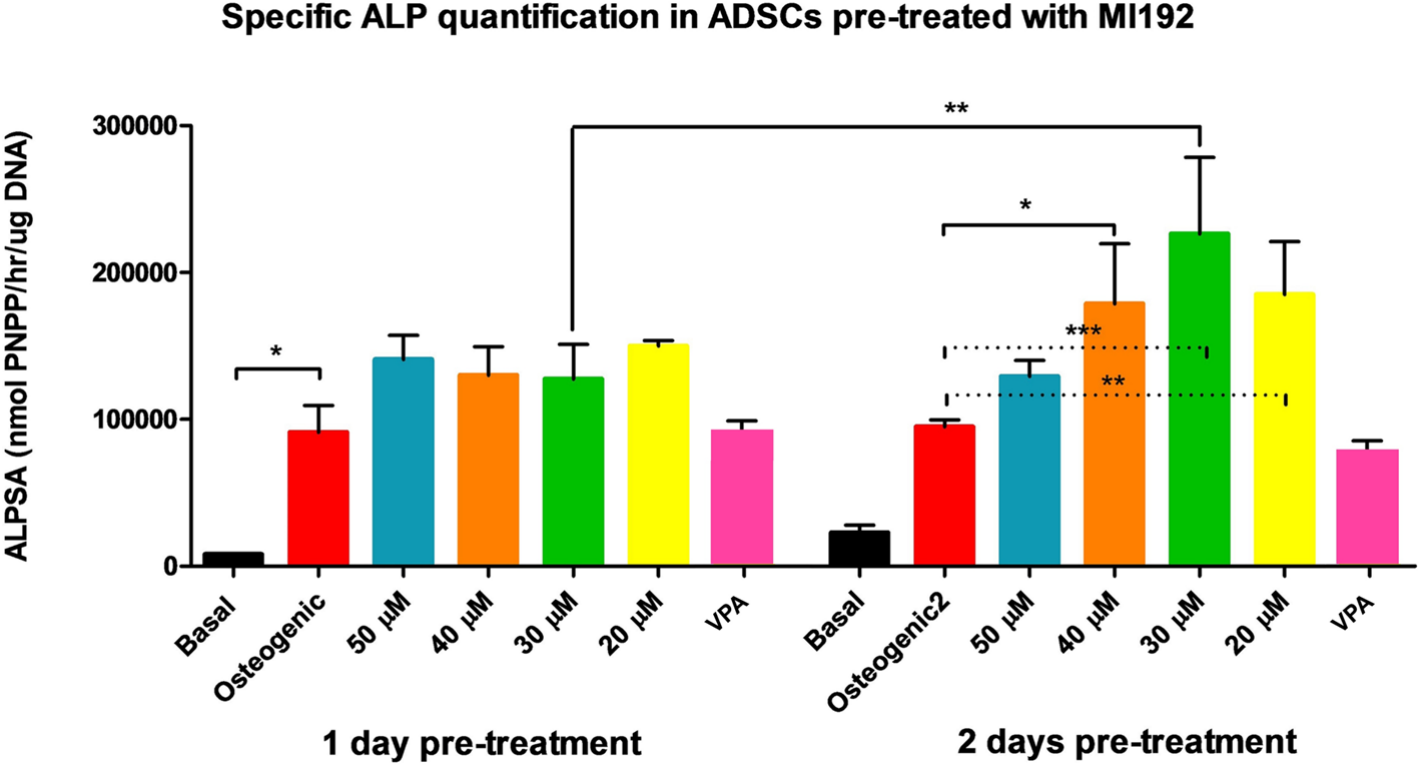
Specific ALP quantification in hADSCs pretreated with MI192 ALP was quantitatively assessed with a biochemical assay and normalised to DNA content, giving the ALPSA. hADSCs were pre-treated with a range (20–50 µM) of MI192 doses for different treatment periods (1 or 2 days); *P≤0.05, **P≤0.01, and ***P≤0.001.

ALP was quantitatively assessed with a biochemical assay and normalised to DNA content, giving the ALP-specific activity (ALPSA). hADSCs were pre-treated with a range (20–50 µM) of MI192 doses for different treatment periods (1 or 2 days). In the 1-day pre-treatment groups, the osteogenic medium positive control group showed a statistically significant increase (*P*≤0.05) in ALPSA over the basal medium negative control group (Figure 6). However, there was no significant difference between the MI192 pre-treated groups and the osteogenic medium positive control or between the test groups (*P*>0.05). In comparison, after 2 days of pre-treatment, all MI192 pre-treated cells have a statistically significant incensement of ALPSA over the osteogenic medium positive control. About 30 µM of MI192 pre-treatment group had the highest level of ALPSA of all groups. In addition, the ALPSAs in both 30 and 20 µM MI192 groups were statistically significantly higher than that of the osteogenic control groups (*P*≤0.001 and *P*≤0.01, respectively). The ALPSAs in both 50 and 40 µM of MI192 groups were statistically significantly higher than that of the osteogenic control groups (*P*≤0.05). Interestingly, when compared between the same pre-treatment concentrations of MI192 over the two different pre-treatment periods, there was a significant enhancement of ALPSA in the cells pre-treated by 30 µM of MI192 for 2 days compared with that of the cells pre-treated with the same MI192 concentration for 1 day (*P*≤0.01).

### MI192 pre-treatment arrested hADSCs cell cycle and induced apoptosis

After pre-treatment for 2 days with 30, 80 µM of MI192, and 2 mM VPA, FACS cell cycle progression analysis results showed that there was a significant reduction of G0G1-phase cells in the group that was pre-treated with 30 µM of MI192 compared with the basal medium control (*P<*0.05). However, there was no significant difference between 80 µM if MI192 pre-treatment group and the basal medium group (*P*>0.05).

In the meantime, there was a significant reduction of cells in the S-phase in the groups that were pre-treated with 30 µM (2.68%), 80 µM (2.58%) MI192, and 2 mM VPA (3.93%) compared with the basal medium control (7.39%). However, there was no significant difference between 30 and 80 µM of MI192 pre-treatment groups (*P*>0.05).

**Figure 7:**
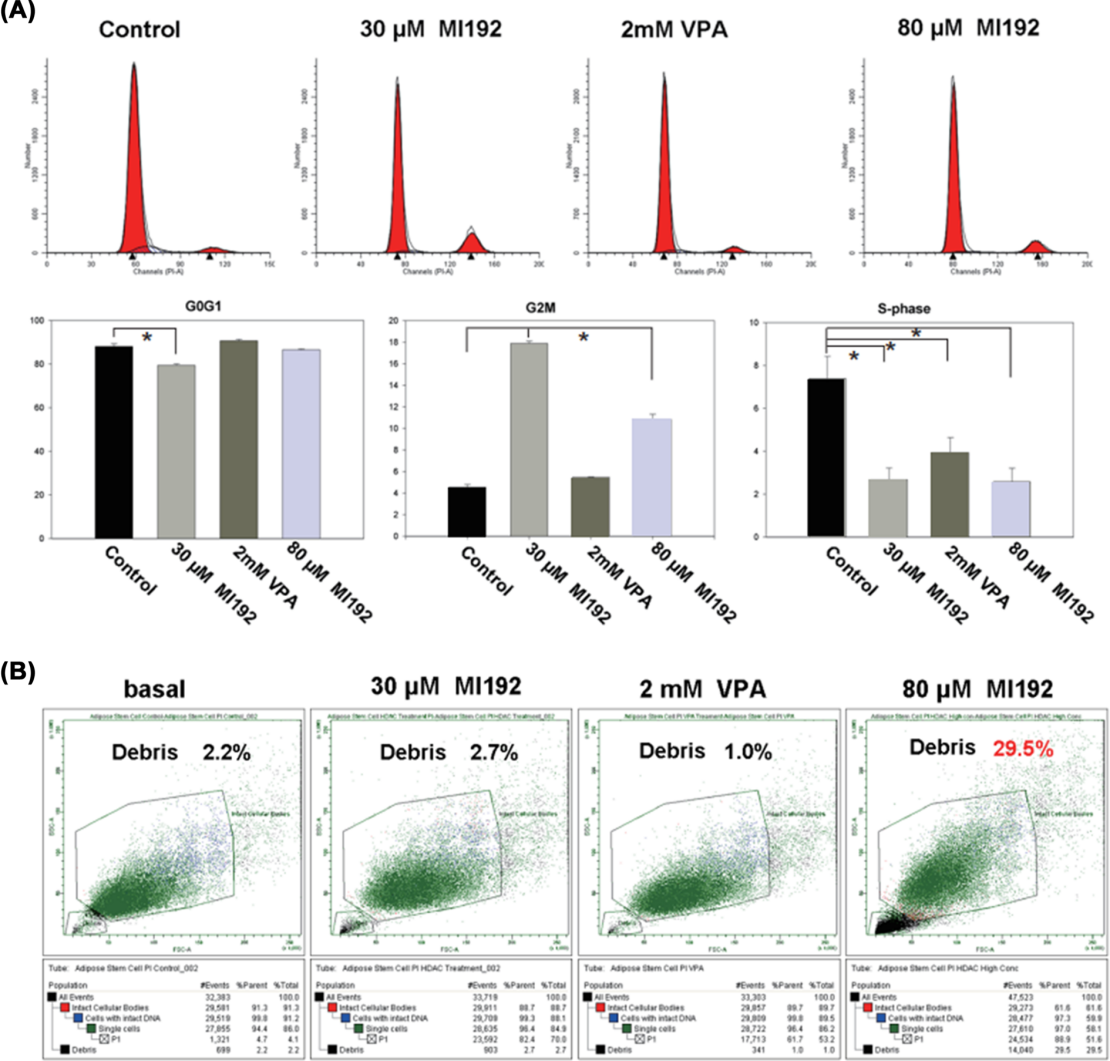
hADSCs treated with MI192 showing cell cycle arrest and reduction of cell viability (**A**) hADSCs treated for 2 days with 30 µM of MI192, 2 mM VPA, 80 µM MI192, and control were analysed by FACS for cell cycle progression. A significant reduction of S-phase cells along with an increase in the number of G2/M cells in the samples that were treated with 30 or 80 µM MI192 as compared with the basal controls. However, with 2 mM VPA treatment, a significant reduction of S-phase cells along with no significant variation in the number of cells in the G2/M or G0G1 phase. The upper half images represent the cell cycle diagram of four groups. (**B**) FACS analyses suggested that 80 µM of MI192 could induce apoptosis or cytotoxicity. Debris was 29.5%. These data revealed a significant increase in the number of apoptotic cells in the cultures that were treated with the high concentrations of MI192 compared with that of the control (*P*≤0.05). *P≤0.05.

In contrast, the groups pre-treated with 30 or 80 µM of MI192 increased the number of G2/M cells compared with that of 2 mM VPA positive and basal medium negative controls (*P*<0.05). There was also a significant increase in cell numbers in G2/M phases in the 30 µM of MI192 pre-treatment group compared with that in the 80 µM of MI192 pre-treatment group (*P*<0.05) (Figure 7A).

FACS analyses showed a higher percentage of debris in the 80 mM of MI192 pre-treatment group (29.5%). In comparison, the 30 µM of MI192 group has much low debris (2.7%), which is similar to the VPA group (1.0%) and the negative control (2.2%) (Figure 7B).

### Effect of MI192 pre-treated on hADSCs osteogenic gene expression

**Figure 8:**
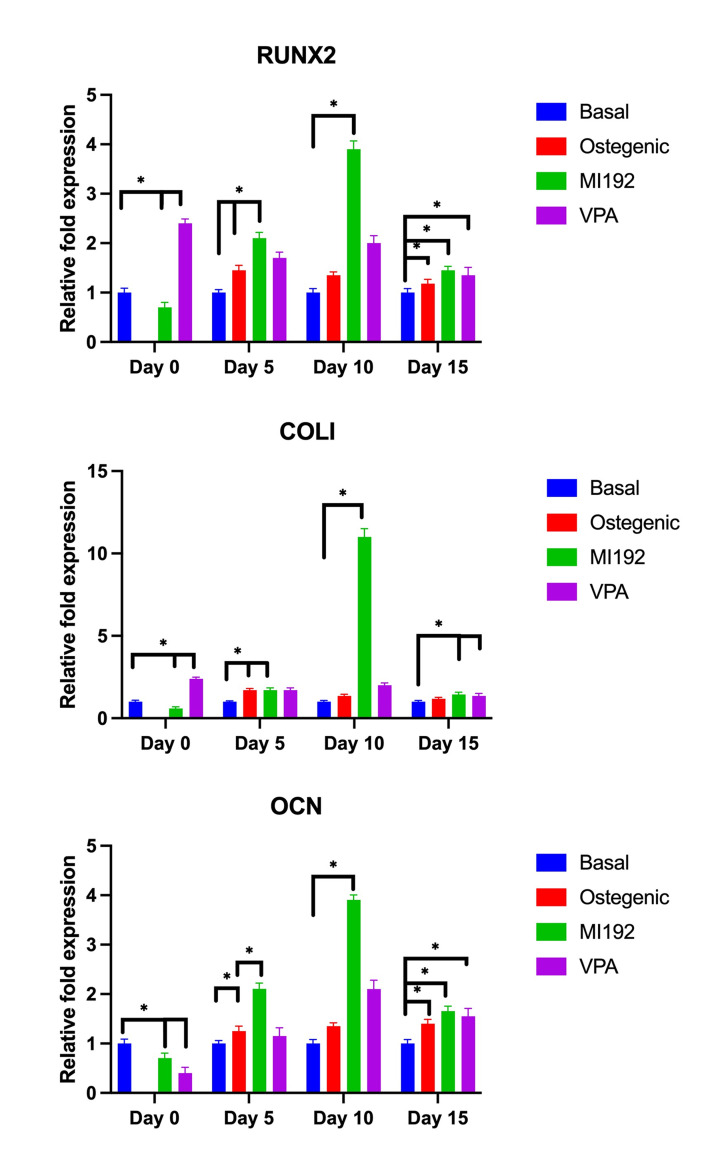
Gene expression in osteogenic induction of MI192 pre-treated hADSCs Real-time PCR analysis further indicated an increased expression of *RUNX2, COL1*, and *OCN* in MI192 and VPA groups, as compared with non-induced controls. There was an 0.5- to 2.5-fold increase in the mRNA levels of *RUNX2* in the cells obtained from MI192 pre-treated group versus only the osteogenic group during the osteogenic induction 5 or 10 days. Furthermore, the mRNA level of *COL1* was higher in MI192 pre-treated group than in other groups after 10 days of osteogenic induction. Moreover, the mRNA level of *OCN* was higher in MI192 pre-treated group than only the osteogenic group during the osteogenic induction of 5 or 10 days. However, MI192 treatment significantly up-regulated the gene expression of *RUNX2*, *COL1*, and *OCN* compared with that of the basal medium along control group (*P*<0.05) at day 15; *P≤0.05.

Quantitative real-time PCR analysis (Figure 8) showed that MI192 pre-treatment for 2 days (day 0) down-regulated the gene expression of *RUNX2*, *COL1*, and *OCN* compared with VPA (*P*<0.05, respectively) and basal medium alone controls (*P*<0.05, respectively). In comparison, VPA treatment for 2 days significantly up-regulated *RUNX2* (*P*<0.05) while down-regulating *COL1* and *OCN* gene expression (*P*<0.05) compared with the basal medium group.

As expected, at 5 days, the cells in OS for 5 days had up-regulated the gene expression of *RUNX2*, *COL1*, and *OCN* compared with basal medium (*P*<0.05) alone (Figure 8). MI192 pre-treatment caused significant up-regulation of *RUNX2* and *OCN* compared with the OS groups (*P*<0.05). It also caused significant up-regulation of *COL1* gene expression compared with the basal medium alone group (*P*<0.05). However, there was no significant difference in *COL1* gene expression (*P*>0.05) between the MI192 pre-treatment group and the two controls of VPA pre-treatment and OS without MI192 pre-treatment.

On day 10, MI192 pre-treatment resulted in significant up-regulation of *RUNX2*, *COL1*, and *OCN* compared with the basal medium groups (*P*<0.05). There was a 4-fold increase for *RUNX2*, an 11-fold increase for *COL1*, and a 4-fold increase in *OCN* expression when comparing the MI192 pre-treatment group and the basal medium alone group. In contrast, the VPA pre-treatment group had only about 2-fold increase in all three genes compared to basal medium control.

On day 15, cells pre-treated with MI192 significantly up-regulated gene expression of *RUNX2*, *COL1*, and *OCN* compared with the basal medium control (*P*<0.05). Similarly, both the VPA pre-treated group and OS culture group had significantly up-regulated expression of *RUNX2*, *COL1*, and *OCN* compared with the basal medium control (*P*<0.05). However, there was no significant difference between the MI192 pre-treated group and the positive control VPA and OS groups (*P*>0.05).

### *Effect of MI192 pre-treatment on* RUNX2 *and* OCN *proteins and mineralisation*

**Figure 9:**
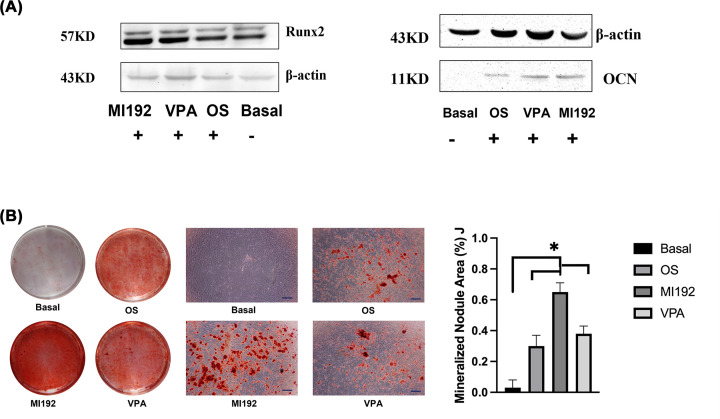
Effect of 30 μM of MI192 pre-treatment for 2 days on the expression of RUNX2 and OCN proteins in hADSCs (Western blot) and calcium accumulation at day 21 (Alizarin red) (**A**) Western blot: the expression of RUNX2 (left) and OCN (right) in hADSCs, and representative bands of the proteins were shown in each panel. Pre-treatment with 30 μM of MI192 led to significantly increased expression of RUNX2 and OCN compared with other groups (*P*<0.01, respectively). (**B**) Alizarin Red staining and semi-quantitative assay: Calcium deposition at day 21 (scale bar: 100 μm); (*P*≤0.05). ^+^*P*<0.01, *P≤0.05.

Western blot was run to detect the expression of RUNX2 and OCN in hADSCs, and representative bands of the proteins were shown in each panel. Pre-treatment with 30 µM of MI192 led to increased expression of RUNX2 and OCN significantly compared with other groups (+*P*<0.01, respectively) (Figure 9A). The effects of MI192 pre-treatment on hADSCs extracellular matrix calcium deposition were identified via Alizarin red staining. MI192 substantially enhanced hADSCs alizarin red staining for calcium deposition compared with that in the basal medium culture and the other two groups (Figure 9B). Following semi-quantitative analysis, it was confirmed that the MI192 pre-treated group exhibited a significant increase in the mineralised nodule areas of four groups after 3 weeks (*P<0.05).

## Discussion

It is well understood that there is a major clinical need for innovative approaches to the restoration of bone structure and function [68,69]. Bone tissue engineering seeks to address this, and it has been generally accepted that bone tissue engineering requires three basic elements: cells, biomaterial scaffolds, and growth factors [70–72]. Although there is an increasing trend to consider mechanical simulation and the microenvironment as a fourth element in functional tissue engineering [73,74].

As discussed earlier, human adipose-derived stem cells are one of the most attractive stem cell sources for bone tissue regeneration [75–77]. Although many different protocols have been developed for controlling cell proliferation and differentiation [58,78], it is still a massive challenge to find methods that are simple, safe, cost-effective, and mimic biological/physiological processes of controlling stem cell behaviour for clinical applications. The present study was undertaken to investigate if altering a cell’s epigenetic makeup can aid in the control of stem cells, with osteogenic differentiation as a long-term goal. We have examined the effect of an HDAC2/3 selective inhibitor MI192 on hADSCs proliferation and osteogenic differentiation *in vitro*.

In the present study, the population doubling time of hADSCs at passage 5 in basal medium and OS on normal cell culture plastic was 42 and 50 h, respectively. As expected, osteogenic culture increased the cell doubling time, and this was within the ranges reported in the literature [79]. However, when the cells were cultured in 1, 10 µM of MI192, the PDTs were further increased to 80 and 90 h, respectively. However, a higher concentration of MI192 and a longer treatment period showed a cytotoxic effect on the hADSCs. After 3 days of pre-treatment, even 1 µM of MI192 group was also significantly more cytotoxic than the control group (*P*≤0.01) (Figure 2). This is similar to our previous studies that MI192 affected hADSCs morphology and viability [47].

By way of explanation for this, when the cell cycle progression of the cells was analysed, we discovered that MI192 (30 and 80 µM) treated cells were paused at the G2/M phase of the cell cycle, along with having a reduction of S-phase cells, compared with untreated and VPA treated controls. These results indicated that MI192 pre-treatment plays a key role in controlling the cell cycle [80], similar to various *in vitro* and *in vivo* studies in the literature [18,81], It has long been known that HDACis causing cell cycle arrest is a mechanism which acts as anti-cancer therapeutic, arresting cell growth, for example, in acute promyelocytic leukaemia and cell lines derived from colon, lung, and prostate carcinomas [82] and cell lines derived from neuroblastoma, glioma, and teratocarcinoma [83]. Studying this mechanism further, Li et al. (2015) showed that HDAC10 regulates the G2/M phase transition in the cell cycle via a novel let-7*-*HMGA2*-*cyclin A2 pathway, and cyclin A2 overexpression rescues the HDAC10 knockdown-induced G2/M transition arrest [18]. Previous reports show that HDAC10 interacts with HDAC3 but not with HDAC4 or HDAC6 [84]. Therefore, the inhibition of HDAC2/3 by MI192 may interfere with HDAC10 and prevent the G2/M cell cycle transition. Different HDACs and cell types have resulted in cell cycle arrest at different points in the cell cycle, with the results from others showing, for example, cervical carcinoma cells arresting at G1 [85] or human lymphatic endothelial cells at G0/G1 [86]. Although the mechanisms behind this are still not very clear, the effect of MI192 on the cell cycle could help to synchronise the hADSCs, and a less compacted DNA structure could help with the gene transcription procedure during the osteogenic induction.

This could be due to the cell cycle halting effect, as a cell’s natural recovery system may take over, and apoptosis may occur [47,87]. Similarly, Paradis et al. (2015) reported that MS275 (a class I HDACi) and Sirtinol (a class II HDACi) were embryotoxic and teratogenic [34,88]. However, most studies into HDACis and non-cancerous cells are silent on any cytotoxic effects of the HDACis.

Interestingly, after 5 days of culture in 1 and 10 µM MI192 (in basal medium), the inhibition of hADSCs proliferation was in a dose-dependent manner. The MI192 also reduced the positive ALP staining of hADSCs compared with the OS positive control group and the basal medium negative control group. As ALP is a well-known and extensively studied earlier-stage osteogenic marker, these results mean that long-term exposure to MI192 could reduce the osteogenic potential of the exposed cells. The other reports also suggested that ALP plays a critical role in calcified/mineralised tissue formation by regulating phosphate transport [89,90].

Considering both the cytotoxic and osteogenic reduction effects of MI192, a pre-treatment strategy has been developed to optimise the best concentration and period of pre-treatment. This approach was similar to that reported previously in the literature [47,87].

While there is still some cell death from 2 days of pre-treatment with MI192 (Figure 2), there was a reduction of the cytotoxicity of MI192, which is offset by a drastically increased osteogenic differentiation potential of hADSCs. Flis et al. (2009) showed that entinostat (MS275) and suberic bishydroxamate (SBHA) enhanced cytotoxicity [91]. Chidamide synergistically enhanced cytotoxicity in pancreatic cancer cells [92]. This was evidenced by enhanced ALP positive staining and quantitative ALPSA (Supplementary Figures S5 and 6). This effect of MI192 on ALP activity was dose-dependent, and the optimal concentration of MI192 for the pre-treatment of hADSCs was found to be 30 µM with an optimal pre-treatment duration of 2 days. Therefore, these results indicated that the 2 days pre-treatment with MI192 can enhance the osteogenic differentiation of hADSCs. Other groups have reported that the treatment with hDACs can increase stem cells’ osteogenic potential. For example, Cho et al. (2005) used VPA, a non-selective HDAC inhibitor, on human mesenchymal stem cells [26], and Xu et al. (2009) used sodium butyrate and VPA to treat mouse hADSCs [93].

As VPA is known to promote MSCs osteogenic differentiation [26], therefore, it was used as a control in some of these experiments, with the effect of VPA pre-treatment compared with MI192 pre-treatment. Importantly, MI192 was shown to drastically increase the osteogenic potential of cells when compared with VPA (Figure 8). This is an exciting result, as it shows that MI192 has a greater potential than VPA, an HDACi often reported in the literature as being a strong inducer of osteogenic differentiation in stem cells.

As well as ALP activity, MI192’s effect on osteogenic gene expression was explored, including *RUNX2* (an osteogenic transcription factor), *COL1* (a mid-stage osteogenic marker), and *OCN* (a late osteogenic marker) gene expression was measured. Two days of pre-treatment with MI192 (30 μM) prior to osteogenic culture in resulted in an initial (day 0) reduction of osteogenic markers *RUNX2*, *COL1*, and *OCN*, whilst similar VPA treatment significantly up-regulated *RUNX2* but down-regulated *COL1* and *OCN* gene expression compared with basal medium group. This may demonstrate that MI192 has a slower mechanism of action than VPA. After further culture of the cells in the osteogenic medium (shown in 5 and 10 days), the hADSCs pre-treated with 30 μM of MI192 significantly up-regulated osteogenic marker expression when compared with the controls. The spike in osteogenic gene expression was reduced by day 15 when the results were comparable to the controls. This indicates that the MI192 pre-treatment has increased the osteogenic capacity of the hADSCs. Given that MI192 is an HDAC3 (along with HDAC2) specific inhibitor, the fact that HDAC3 is depleted during osteogenic differentiation is interesting and relevant. For example, this reduction in HDAC3 during osteogenic differentiation could indicate that HDAC helps maintain cells in a pluripotent state, and by inhibiting HDAC3, the cell is then more susceptible to the induction of differentiation of cells (e.g., osteogenic shown here).

In the present study, the effects of MI192 pre-treatment on the expression of osteogenesis-related genes and proteins were also investigated. Our finding showed that MI192 significantly up-regulated hADSCs *RUNX2* and *OCN* expression levels and mineralisation throughout the osteogenic culture. It is well known that *RUNX2* is the osteoblastic transcription factor that plays a key role in osteogenic differentiation [94,95]. HDAC1, HDAC3, HDAC4, and HDAC6 are known to inhibit RUNX2 activity in different pre-osteoblastic cell lines and bone-marrow stem cells [96,97]. These quantitative rtPCR results show that whilst MI192 pre-treatment initially down-regulated expression of *RUNX2*, after prolonged culture, the levels of gene expression spiked.

Although the mechanism is still not fully understood, the initial arrest of the cells at the G2M phase of the cell cycle may play some important roles. After the removal of MI192, more cells from the G2M phase entered into the mitotic phase and cytokinesis phase at the same time and response to the osteogenic induction, which, in turn, started the cascade event for osteogenic differentiation into more osteoblastic cells (express stronger ALP). This may be due to cell synchronisation, which brings cells at different stages of the cell cycle into the same phase and enhances the ALP activities of the cells as a whole, improving the efficacy of bone tissue regeneration [98,99].

## Conclusion

The present study has demonstrated that the specific inhibition of HDAC2/3 can achieve epigenetic reprogramming of hADSCs resulting in promoting stem cell's osteogenic differentiation potential. This effect of MI192 is stronger than broadspectrum HDACis, such as VPA. As the epigenetic approach does not change the genome of the stem cells, it is considered safer and more cost-effective to use MI192 for clinical bone repair and regeneration.

## Supplementary Material

Supplementary Figures S1-S6

## Data Availability

The data generated during the current study are available from the corresponding author on reasonable request.

## Open Access

Open access for this article was enabled by the participation of University of Leeds in an all-inclusive *Read & Publish* agreement with Portland Press and the Biochemical Society under a transformative agreement with JISC.

## Abbreviations

ALP: alkaline phosphatase
ALPSA: ALP-specific activity
hADSC: human adipose-derived stem cell
HAT: histone acetyltransferase
HDAC: histone deacetylases
IL-6: interleukin-6
MSC: mesenchymal stem cell
PBMC: peripheral blood mononuclear cells
RA: rheumatoid arthritis
SBHA: suberic bishydroxamate
TNF: tumour necrosis factor
VPA: valproic acid
