# Supplementary material for: The effect of epigenetic reprogramming using MI192 HDAC inhibitor on enhancing the osteogenesis of human adipose-derived stem cells in vitro
Source: Biosci Rep. 2023 Apr 28;43(5):BSR20221635. doi: 10.1042/BSR20221635 (PMC10154459; doi:10.1042/BSR20221635)
Supplement: Supplementary Figures S1-S6 [file BSR-2022-1635_supp.pdf]

Supplementary figures

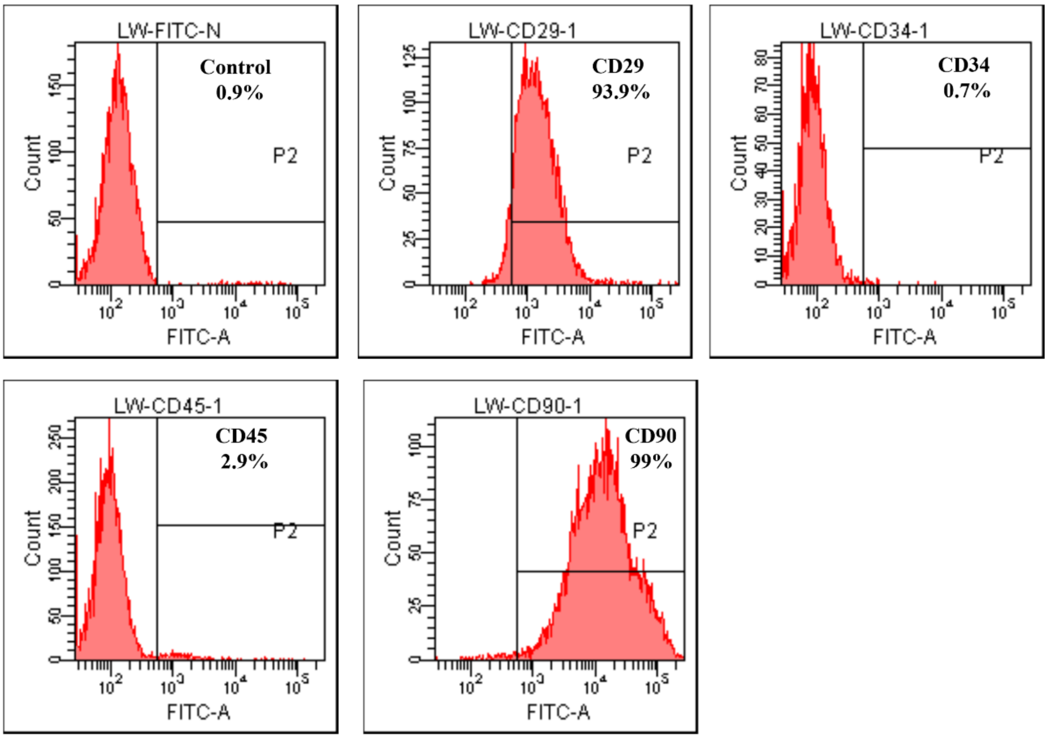

Figure S1: Flow cytometric analysis of hADSCs. hADSCs (P5) revealed expression of CD29 (93.9 %), CD34(0.7%), CD45(2.9%) and CD90 (99 %).

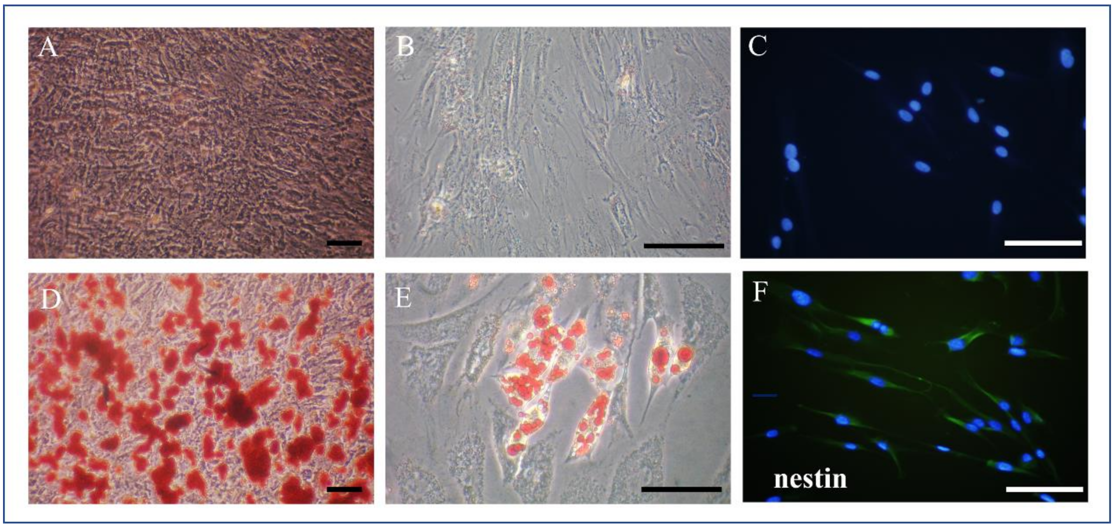

Figure S2: Multilineage differentiation of hADSC

A-C) basal medium culture; D) osteogenic culture; E) adipogenic culture; F) neurogenic induction culture. A & D) Alizarin red staining: red colour shows the positive stain of Alizarin red, which indicates calcium accumulation; B & E) Oil red O staining: red colour shows the positive staining for lipid clusters; C & F) Nestin staining: green colour indicated the positive stain for nestin (neurogenic marker) and the blue colour are nuclei. Scale bar = 50 um.,

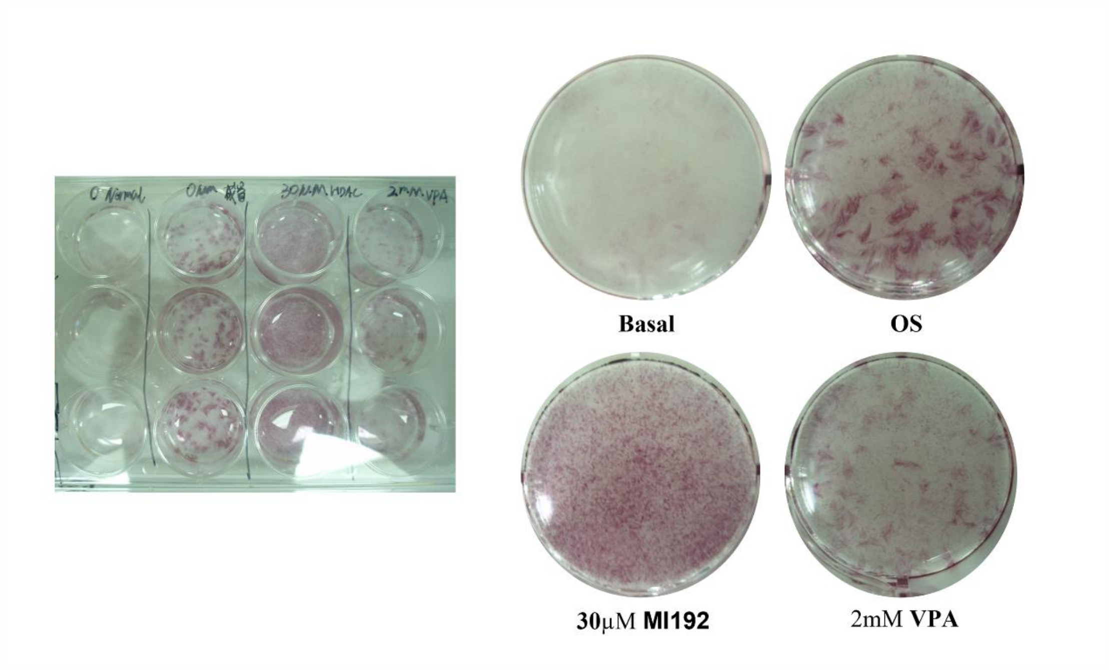

Figure S3: ALP staining on another batch of hADSCs pre-treated with MI192 for 2 days and osteogenic induction for 5 days. ALP staining showed that the group that was pre-treated with 30 μM MI192 for 2 days markedly increased the ALP stain intensity compared to that in the control groups.

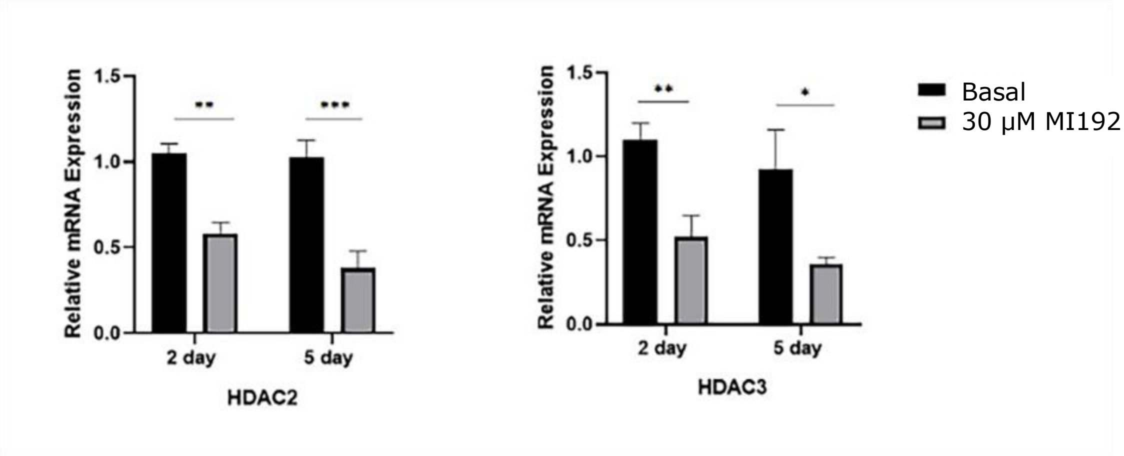

Figure S4: 30 µM MI192 decreased H DAC2 and HDAC3 expression in hADSCs. Mean ± SD (n=3). * $p \leq 0.05$; ** $p \leq 0.01$; *** $p \leq 0.001$. After 2 days and 5 days of pre-treatment with 30 µM MI192, relative mRNA expression of HDAC2 and HDAC3 in hADSCs significantly decreased compared to the control group in basal medium culture.

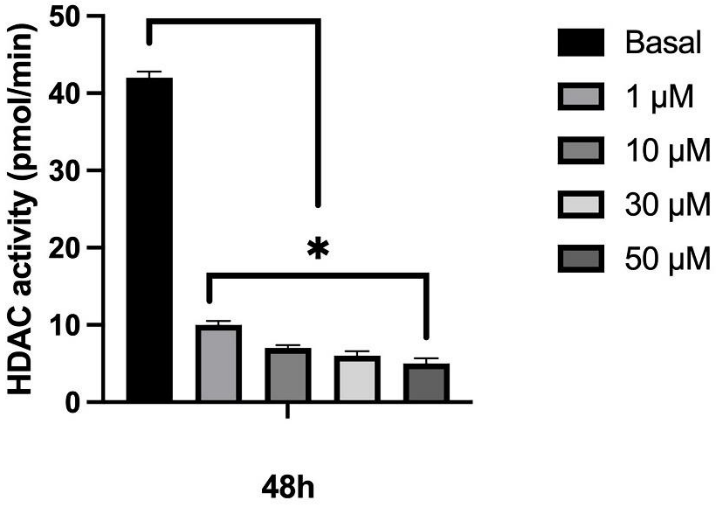

Figure S5: HDAC activity levels of hADSCs after 48h of treatment with different concentrations of MI192. The graph showed the influence of MI192 on hADSCs epigenetic functionality using HDAC activity levels. Treatment with MI192 significantly reduced hADSCs metabolic activity compared to untreated groups. Mean ± SD (n=3). * $p \leq 0.05$.

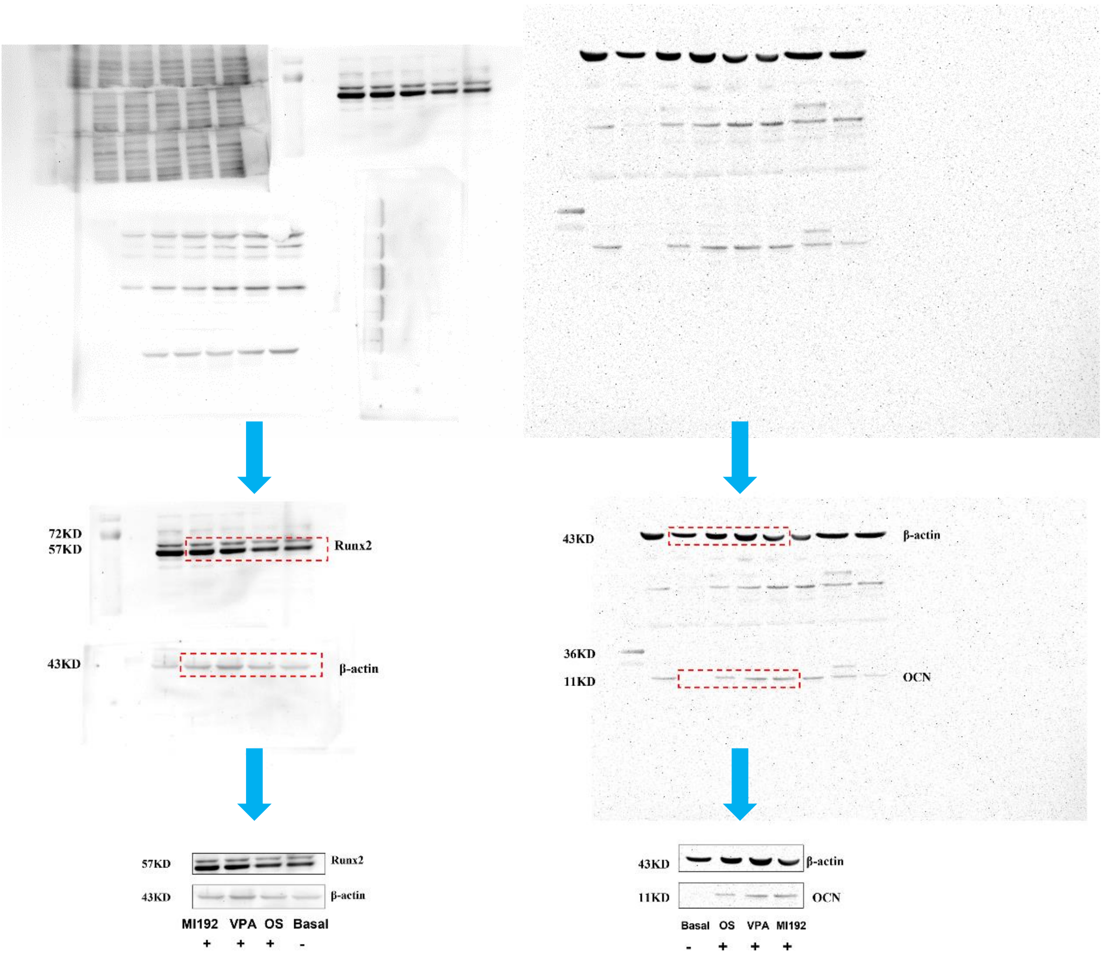

Figure S6: Full uncropped and unedited version sof the Western blot (top panel). The red doted outlines (midle panel) indicated the lanes used to build the figure 9A (bottom panel).
